# Genotype x environment interaction for stomata traits in a rice recombinant inbred population

**DOI:** 10.3389/fpls.2026.1953117

**Published:** 2026-10-30

**Authors:** Afeez Oluniyi Shittu, Glenn B. Gregorio, Tonette Laude, Eureka Teresa M. Ocampo, Antonio G. Lalusin, Eliza Vie Ada, Ramaiah Venuprasad

**Affiliations:** 1 International Rice Research Institute (IRRI), Los Baños, Laguna, Philippines; 2 Institute of Crop Science, College of Agriculture and Food Science (CAFS), University of the Philippines Los Baños (UPLB), College, Laguna, Philippines

**Keywords:** drought stress, genotype × environment interaction, grain yield, moisture regime, recombinant inbred lines (RILs), stomatal traits

## Abstract

Drought is a major abiotic constraint limiting global rice (*Oryza sativa* L.) production. While stomatal traits are linked to drought adaptation, their environmental stability and breeding utility are poorly understood. This study evaluated 272 recombinant inbred lines (RILs) derived from an IRRI 154/NERICA 4 cross under well-watered and reproductive-stage drought-stress conditions across four seasons (2022WS - 2024DS) to quantify genotype-by-environment (G×E) interactions for four stomatal characteristics (density, area, length, and width) on both leaf surfaces. Significant genotypic variation and pronounced transgressive segregation were observed for all traits. While reproductive-stage stress reduced mean grain yield by 84.6%, stomatal density remained numerically stable across moisture regimes. Conversely, cell dimensions exhibited high plasticity, driving a 20.5% reduction in abaxial stomatal area and a 17.2% reduction in length to facilitate rapid closing kinetics. Combined multi-environment analysis revealed that while raw grain yield heritability dropped to 0.39 under stress due to seasonal masking, abaxial stomatal density and area preserved exceptionally robust combined heritability (H^2^ ,  0.92 and 0.99, respectively). Stomatal traits displayed weak direct correlations with final grain yield, indicating they function as upstream components of stress resilience rather than direct drivers of sink capacity. Crucially, these findings demonstrate that breeders can focus exclusively on abaxial stomatal density under well-watered conditions as a unified, high-heritability proxy for large-scale germplasm screenings. This operational shortcut captures baseline genetic variation while completely avoiding stress-induced leaf deformations, offering a highly scalable, high-throughput pathway to accelerate climate-resilient rice development.

## 1 Introduction

Rice (*Oryza sativa* L.) is the primary staple food for more than half of the world’s population, providing the majority of daily caloric intake across Asia, Africa, and Latin America (Khush, 2013; Mishra et al., 2021). Global rice demand is projected to increase substantially by 2050 in response to population growth, urbanization, and shifting dietary patterns, placing considerable pressure on production systems already challenged by climate variability (FAO, 2018). Among the abiotic stresses limiting rice productivity, drought is the most widespread and economically damaging, affecting approximately 45% of the global rice area under rainfed conditions and causing yield losses ranging from 17 to 40% in moderate drought years, and exceeding 50% during reproductive stage stress (Jarin et al., 2024; Sadhukhan et al., 2024).

Drought stress impairs a wide range of physiological and biochemical processes in rice, including leaf water status, photosynthetic efficiency, nutrient uptake, and reproductive development (Farooq et al., 2024). Among the morpho-physiological traits mediating plant responses to water deficit, stomatal regulation occupies a central position, directly governing the trade-off between carbon assimilation and transpirational water loss (Lawson and Matthews, 2020). Stomata, a specialized epidermal structure comprising a pair of guard cells and associated subsidiary cells, regulate the diffusion of CO_2_ into the leaf mesophyll and water vapor out of the plant, thereby determining both photosynthetic capacity and water-use efficiency (WUE) (Hetherington and Woodward, 2003; Raven, 2014). Under drought conditions, stomatal closure is one of the earliest and most rapid adaptive responses, restricting transpirational water loss and preventing hydraulic failure, yet simultaneously limiting CO_2_ uptake and suppressing photosynthesis (Chaves et al., 2016; Hassan et al., 2023).

Stomatal traits including stomatal density, size, aperture, and conductance (gs) are highly variable among rice genotypes and have been increasingly recognized as important selection criteria for drought adaptation and yield stability (Caine et al., 2018; Phunthong et al., 2024). Reduced stomatal density has been associated with improved WUE and enhanced drought tolerance, while intermediate gs values that maintain CO_2_ assimilation during stress have been linked to better yield performance under water-limited conditions (Caine et al., 2023; Qiao et al., 2024). However, stomatal traits are said to be quantitatively inherited and genotype-by-environment interaction (GXE) is poorly understood, complicating their reliable deployment in breeding programs (Pitaloka et al., 2022; Fernado et al., 2025).

Despite growing recognition of the importance of stomatal traits in drought adaptation, quantitative dissection of stomatal characteristics and their relationship with yield stability across environments remains limited in rice RIL populations. A comprehensive understanding of rice stomatal traits (density, area, length, and width) on both adaxial and abaxial leaf surfaces, their interrelationships, interaction with yield and related traits under drought and well-watered field conditions in multiple years is therefore essential in determining breeding strategies drought tolerance in rice.

The present study aimed to study the stomatal traits and evaluate yield stability in a rice RIL population assessed under contrasting drought stress and non-stress field environments over multiple seasons. Specifically, this study sought to characterize variation for stomatal traits among RILs in well-watered and drought stress conditions, examine associations between stomatal traits and yield components, and quantify GEI effects using GGE analyses.

## 2 Materials and methods

### 2.1 Planting materials and mapping populations development

A biparental mapping population was developed from a cross between IRRI 154 and NERICA 4. IRRI 154 (female parent) (released in the Philippines as NSIC Rc222) is a high-yielding lowland rice cultivar with moderate susceptibility to drought stress, whereas NERICA 4 (male parent) is a drought-tolerant upland rice variety widely cultivated across Africa. The F_1_ plants were self-pollinated to generate a large F_2_ population comprising approximately 2,500 individuals, thereby maximizing genetic recombination and allelic segregation. Individual F_2_ plants were advanced through single-seed descent, and 311 F_3_ progenies were randomly selected for further selfing. During the F_3:4_ generation, the population was reduced to 272 families following the removal of lines exhibiting extreme phenotypes for plant height and late flowering. The remaining families were subsequently advanced by continuous selfing to the F_4:5_ generation (RILs), which was used for phenotypic evaluation. An overview of the experimental workflow and phenotyping pipeline is presented in **Figure 1**.

**Figure 1:**
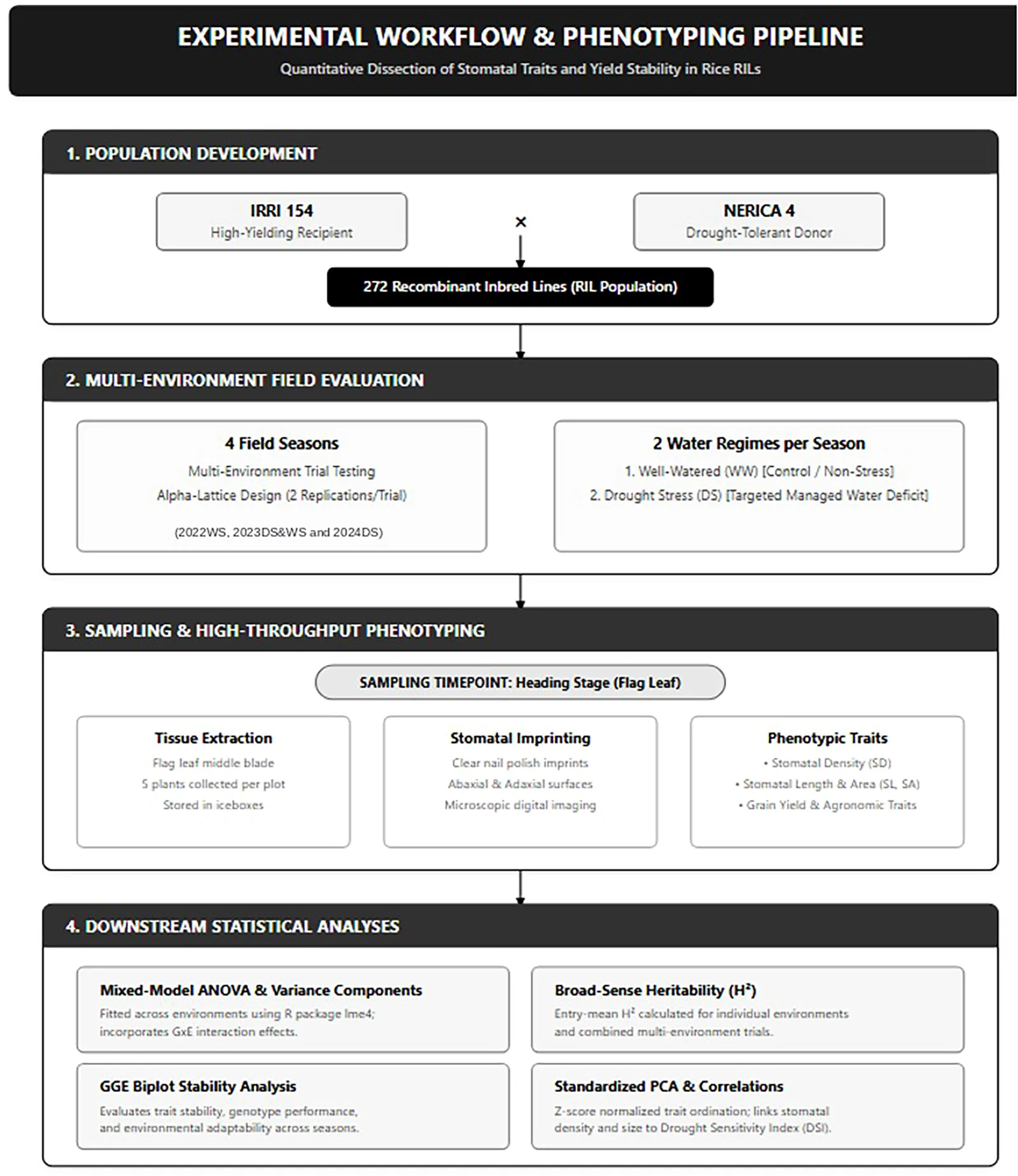
Schematic diagram of the experimental workflow and analytical framework. The study evaluated 272 recombinant inbred lines (RILs) derived from a cross between IRRI 154 and NERICA 4 across four field seasons under two contrasting water regimes (Well-Watered, WW; Drought Stress, DS). Flag leaf samples were collected at the heading stage to extract stomatal imprints for quantitative traits dissection, followed by integrated statistical and multivariate yield stability analyses.

This study was conducted at the experiment station of the International Rice Research Institute (IRRI), Los Baños, Laguna, Philippines, from 2022 wet season (WS) through 2024 dry season (DS). IRRI is located at 14°13’ N latitude, 121° 15’ E longitude, and at an elevation of about 21 m above mean sea level. The soil type is a Maahas clay loam, isohyperthermic mixed typic tropudalf.148139 N latitude, 1218159 E longitude at an elevation of about 21 m above mean sea level.

### 2.2 Phenotyping

The RILs, parents, and checks were phenotyped both under well-watered irrigated trials (control) and drought stress trials across four seasons: 2022WS, 2023DS, 2023WS and 2024DS. Checks included were DRR42, NERICA3, and IR74371-54-1-1. The seeds were sown in seed beds and after 21 days, seedlings were transplanted into well-prepared levelled paddy fields under non-stress control (NS) and drought stress (DS) following 20 x 20 cm spacing with 2 seedlings per hill in a row. A single row of 3 m length was used for all the trials.

The experiments were all conducted in an alpha lattice design (2 reps and 5 entries per block) with six checks including the two parentals. After transplanting, around 5 cm of standing water was preserved in the field until drainage took place before the harvest. Basal inorganic fertilizer was applied at the rate 30-30–30 kg NPK ha^-1^ while topdressing was done in 2 splits, at maximum tillering and booting stage at 30 kg N ha^-1^. Weeds were controlled by chemical applications whenever we noticed high weed pressure on the experimental areas. Standard pest and diseases control measures applicable at IRRI farm were applied. Birds were controlled by covering the trials with net at the reproductive stage.

The drought trials were conducted in a field with a rainout shelter automated to cover the plots during rain. These trials were managed like the irrigated trials in all aspects from seeding stage to 30 days after transplanting. Drought stress was introduced during the reproductive stage at 30 days after transplanting (DAT) by draining the paddy and withholding irrigation until the soil moisture tension reached -70 kPa at a depth of 20 cm using tensiometer. At this moisture level, severe leaf rolling and firing were observed. The fields were then re-flooded and drained again after approximately 24 hours. Every time the soil moisture reached -70 kPA, the cycle of re-flooding and draining the field was repeated until harvesting. This stress-imposition protocol was developed and routinely applied at IRRI (Bernier et al., 2007; Venuprasad et al., 2007, Venuprasad et al., 2008).

### 2.3 Data collection and statistical analysis

The study measured phenotypic data (IRRI SES, 2015) such as days to flowering (DFLW), plant height (HT), and grain yield per square meter (GY). Stomata traits, including abaxial stomata density (ABXDS), abaxial stomatal area (ABXAR), abaxial stomatal length (ABXLT), abaxial stomatal width (ABXWT), adaxial stomatal density (ADXDS), adaxial stomatal area (ADXAR), adaxial stomatal length (ADXLT), and adaxial stomatal width (ADXWT) were evaluated under both well-watered and drought stress conditions using five random hills per line. Plants were harvested at 85% physiological maturity, then dried to 14% moisture content and weighed. Relative yield reduction (RYR) under stress was calculated for each genotype as RYR , [1 − (mean performance under stress/mean performance under non-stress)] × 100, following Venuprasad et al. (2007).

Phenotypic data collected from the population under WW and DS conditions across seasons were then analyzed using R software version 4.6.0 (R Core Team, 2026). Individual trial analysis was performed using Bioflow software (Fowobaje et al, 2024). Multi-environment trial analyses were performed using the METAN package (Olivoto and Lúcio, 2020). For the analysis, replications, and blocks within replications were considered random. Seasons and treatments (WW and DS) were treated as fixed. Genotypes were considered random and fixed for estimating variance components and means, respectively.

A combined analysis of variance (ANOVA) was performed to evaluate the effects of genotype (G), treatment (T), and their interactions. Significance of the sources of variation was determined using F-tests, and treatment means were separated using Fisher’s least significant difference (LSD) test at the 5% probability level.

Trait distributions across environments were visualized using box plots. Pearson correlation coefficients were calculated to determine relationships among agronomic and stomatal traits using corrplot package in R studio. Correlation matrices were visualized to assess trait associations under contrasting moisture regimes.

Genotype × environment interaction (GEI) was investigated using genotype plus genotype-by-environment (GGE) biplot analysis implemented in the METAN package (Olivoto and Lúcio, 2020). The GGE model was fitted using singular value decomposition of environment-centered data, and the first two principal components (PC1 and PC2) were used to construct biplots following the methodology of Yan and Tinker (2006). The GGE biplots were used to evaluate environmental discrimination, relationships among environments, and the magnitude of genotype × environment interaction. Prior to PCA, all phenotypic traits were standardized (mean-centered and scaled to unit variance) using scale. , TRUE in prcomp(), to prevent traits with larger variance (e.g., stomatal density, grain yield) from disproportionately influencing the principal components (Mohammadi and Prasanna, 2003).

For variance component estimation and mixed-model analyses, linear mixed-effects models were fitted using the lme4 package (Bates et al., 2015) when required. Model diagnostics were assessed through residual and fitted-value plots to verify model assumptions. All graphical outputs, including box plots, correlation heatmaps, and GGE biplots, were generated in R studio (R Core Team, 2026).

Heritability on a plot basis (h^2^_plot_) was estimated as:


$$\frac{\sigma^{2}G}{\left(\sigma^{2}G+\sigma^{2}E\right)}$$

where σ^2^G is the genetic variance among genotypes and σ^2^E is the environmental (residual) variance. For the combined multi-environment analysis, each season × water-regime combination was considered an environment. The model included environment as a fixed effect, genotype as a random effect, genotype × environment interaction as a random effect, and replication and incomplete blocks as random effects nested within environment. The model was.


$$y_{ijkl}=\mu +E_{j}+R_{k\left(j\right)}+B_{l\left(kj\right)}+G_{i}+{\left(G\times E\right)}_{ij}+\epsilon_{ijkl}$$

Combined broad-sense heritability on a genotype-mean basis was calculated as:


$$H^{2}=\frac{\sigma_{G}^{2}}{\sigma_{G}^{2}+\frac{\sigma_{GE}^{2}}{e}+\frac{\sigma_{\epsilon }^{2}}{re}}$$

where σ^2^G is the genotypic variance, σ^2^GE is the genotype × environment interaction variance, σ^2^ϵ is the residual (error) variance, e is the number of environments, and r is the number of replications per environment.

Broad-sense heritability represents the proportion of the total phenotypic variance that is attributable to genetic differences among genotypes.

#### 2.3.1 Stomatal extraction and analysis

On heading day, flag leaves were collected from five randomly selected hills in each plot across the replicates. To preserve sample quality, leaf samples were collected from the field, stored in an ice box, and transported to the laboratory prior to imprint preparation. A thin layer of transparent nail polish was applied to an approximately 5 cm^2^ area in the middle of the leaf, covering both the adaxial and abaxial surfaces, to obtain stomatal imprints. After the nail polish was dried, an adhesive tape was carefully applied and peeled off to collect the stomatal impressions, according to the method of Kusumi (2017).

The imprints were analyzed by pasting each imprint on a glass slide and observing at 20x magnification using a Zeiss Axioplan 2 with a Nikon DS-Ri2 imaging system. Imaging and analysis were conducted on three representative sections for each imprint using ImageJ v.1.49b (Schneider et al., 2012). In total, about 20,000 images and about 100,000 individual stomata were measured.

## 3 Results

Clear phenotypic variation among the RILs was observed under drought and well-watered conditions across the seasons for the traits. Generally, the susceptible parent IRRI 154 showed inferior performance for yield while the tolerant parent NERICA 4 showed superior performance across the seasons under drought stress. In contrast, some of the RILs outperformed tolerant parent NERICA 4 under drought stress for grain yield and other agronomic traits across the four seasons. At least 70% of RILs showed better performance than the susceptible parent.

### 3.1 Phenotypic performance of rice RILs, parents, and checks for agronomic traits under drought stress and well-watered conditions.

Considerable phenotypic variation was observed among the RILs for agronomic and stomatal traits evaluated under well-watered (WW) and drought stress (DS) conditions across four seasons (2022WS, through 2024DS) (**Supplementary Tables 1a, b**; **Table 1**). Significant differences were observed among genotypes for days to flowering, plant height, grain yield, yield reduction ratio (YR), and stomatal traits measured on both abaxial and adaxial leaf surfaces, indicating the presence of considerable genetic variability within the population.

**Table 1:** Combined descriptive statistics of agronomic and stomatal traits of rice RILs under drought stress and non-stress conditions across the seasons.

Genotypes											Abaxial surface											
	Days to flowering		Plant height (cm)			Grain Yield (gm-2)					Density/ mm^2^				Area (μm^2^)		Length (μm)			Width (μm)		
	Non-stress	stress	Non-stress		stress	Non-stress			stress		Non-stress		stress		Non-stress	stress	Non-stress		stress	Non-stress	stress	
IRRI154	89.19	89.92	101.19		72.86	622.17			14.10		1403.46		1402.39		205.24	241.09	21.74		19.34	9.67	13.13	
NERICA4	71.30	70.83	94.34		80.17	346.67			91.88		1028.98		1148.44		191.96	153.50	20.54		17.44	10.00	9.00	
Min	64.35	65.78	65.10		72.94	584.19			8.69		961.59		792.17		149.25	113.49	17.00		13.83	8.75	7.67	
Max	89.75	88.01	118.97		90.56	135.34			126.33		1656.98		1837.10		268.00	275.16	25.13		23.33	11.37	12.00	
Progeny Mean (n, 272)	75.57	76.77	90.29		72.94	308.96			47.54		1249.48		1265.92		208.15	165.43	21.15		17.51	10.04	9.68	
LSD (P<0.05)	2.21	2.54	4.06		3.98	2.91			8.58		38.68		135.38		3.01	7.36	0.54		1.50	0.30	0.82	
H	0.58	0.66	0.75		0.74	0.54			0.39		0.81		0.92		0.82	0.99	0.74		0.87	0.66	0.89	
Adaxial surface																						
	Density/ mm^2^		Area (μm^2^)				Length (μm)			Width (μm)											
Genotypes	Non-stress	stress	Non-stress	stress			Non-stress	stress		Non-stress		stress									
IRRI154	1340.24	1242.54	207.99	198.75			21.83	19.14		9.68		10.78									
NERICA4	903.17	978.05	187.79	154.78			19.87	16.94		9.67		9.39									
Min	895.87	771.83	142.13	116.67			16.75	14.67		8.11		8.33									
Max	1345.04	1381.50	251.25	265.33			24.75	22.17		10.88		11.50									
Progeny Mean (n, 272)	1079.20	1075.02	184.52	169.97			20.13	17.95		9.26		9.62									
LSD (P<0.05)	50.45	112.12	3.34	7.57			0.61	1.42		0.33		0.80									
H	0.80	0.90	0.83	0.99			0.74	0.90		0.62		0.79									

LSD Least significant difference, H heritability (broad sense).

Grain yield was the trait most severely affected by drought stress with differential responses between the two parental genotypes across seasons (**Supplementary Table 1a**). Under non-stress conditions, IRRI 154 consistently produced higher grain yield, averaging at 534.23 g m^-2^ through seasons, compared with 290.69 g m^-2^ for NERICA 4. The highest yield of IRRI 154 was recorded during the 2023DS (868.04 g m^-2^), proving its strong yield potential under optimal conditions. In contrast, NERICA 4 showed a lower average yield but maintained relatively stable performance across seasons. Across seasons under drought stress, IRRI 154 showed yield reductions ranging from 95.9 to 97.7%, whereas NERICA 4 showed comparatively lesser reductions ranging from 39.0 to 77.4%. Within the RIL population, similar responses were recorded where mean yield reduction varied from 75.6% in 2023DS up to 87.1% in 2024DS, showing clear transgressive segregation for grain yield under drought stress. Despite these reductions, several RILs outperformed under drought stress and exceeded the yields of both parental lines. Combined analysis across seasons further confirmed the strong impact of drought stress on grain yield (**Table 1**). RIL mean grain yield decreased from 308.96 g m^-2^ under non-stress conditions to 47.54 g m^-2^ under drought stress, estimated to be an overall reduction of 84.6%. Under drought stress conditions, NERICA 4 produced a higher yield (91.88 g m^-2^) than IRRI154 (14.10 g m^-2^), representing a yield advantage of sixfold, clearly showing noticeably superior drought tolerance of relative to the susceptible parent. As expected, under non-stress conditions IRRI 154 gave greater yield (622.17 g m^-2^) than NERICA4 (346.67 g m^-2^). To identify recombinant inbred lines (RILs) with contrasting performance under differing water regimes, the top five and bottom five RILs were selected based on mean grain yield (GYD) separately under control and stress conditions, and other agronomic traits means are summarized in **Supplementary Table 2**. These results highlight the difference the two parental lines respond to drought and the wider variation present within the RIL population under investigation.

Plant height was also significantly affected by drought stress, yet the level of reduction was quite lower than what was observed for grain yield (**Supplementary Table 1a**). Across conditions, IRRI 154 had greater plant height than NERICA 4. The plant height reduction in IRRI 154 due to stress ranged from approximately 17 to 30% depending on the season. In contrast, NERICA 4 showed minor reductions ranging from 6 to 18%, showing relatively better maintenance of stem growth under water deficit. The RILs mean plant height decreased from 100.30 to 83.21 cm in 2022WS, from 99.43 to 76.06 cm in 2023WS under stress and non-stress conditions, respectively. In 2024DS, a sharp reduction was recorded in RILs, dropping from 77.88 to 53.37 cm under both conditions, respectively. This reflects severe drought-induced inhibition of stem elongation across environments. Combined analysis across seasons (**Table 1**) showed that mean plant height dropped from 90.29 cm under non-stress conditions to 72.94 cm under drought stress, presenting an overall reduction of approximately 19.2%. Although drought stress reduced vegetative growth across the RILs, considerable variation remained, suggesting the presence of genotypes capable of maintaining plant growth under drought conditions.

Days to flowering differed in response to drought stress among seasons and genotypes (**Table 1**). The parental lines showed contrasting flowering period, with IRRI 154 flowering early in 2022WS (-7.65 days, ranging from 94.99 to 87.34 days) and 2023WS (-2.32 days), but delayed flowering in 2023DS (+4.24 days) and most pronounced in 2024DS (+10.57 days, ranging from 81.03 - 91.60 days), reflecting strong seasons x stress interactions probably related to drought timing likely to crop developmental stage. NERICA 4, the early flowering parent (61.42-79.17 days) under well-watered conditions, similarly showed bi-directional responses; delayed flowering in 2022WS (+5.44 days) and 2023DS (+3.98 days), but early in 2023WS (-2.89 days) and 2024DS (-7.91 days). Under drought stress, flowering was late relative to non-stress conditions, although the degree of delay varied among seasons. The progeny mean increased from 74.72 to 76.58 days in 2022WS and from 69.45 to 71.22 days in 2023DS under drought stress. Similar patterns were observed in the remaining seasons as well. Combined analysis across seasons (**Table 1**) showed that mean days to flowering ranged from 75.57 days under non-stress conditions to 76.77 days under drought stress. Even if the shift seemed minor on average, the wide range observed among the RILs indicates strong genetic variation for developmental response to drought. The consistently earlier flowering of NERICA 4 relative to IRRI 154 further suggests contrasting adaptation strategies between the two parental lines. The boxplots of the RIL population across four seasons showed substantial phenotypic variation in stomatal and agronomic traits under well-watered (WW) and drought-stress (DS) conditions (**Figure 2**). For stomatal traits on both leaf surfaces (**Figures 2A-H**), median values were generally lower under DS than WW, with reductions more pronounced for stomatal area and length than for density and width. Similar interquartile ranges between water regimes, together with occasional extreme upper-tail outliers, indicated broad variation and the presence of transgressive segregants.

**Figure 2:**
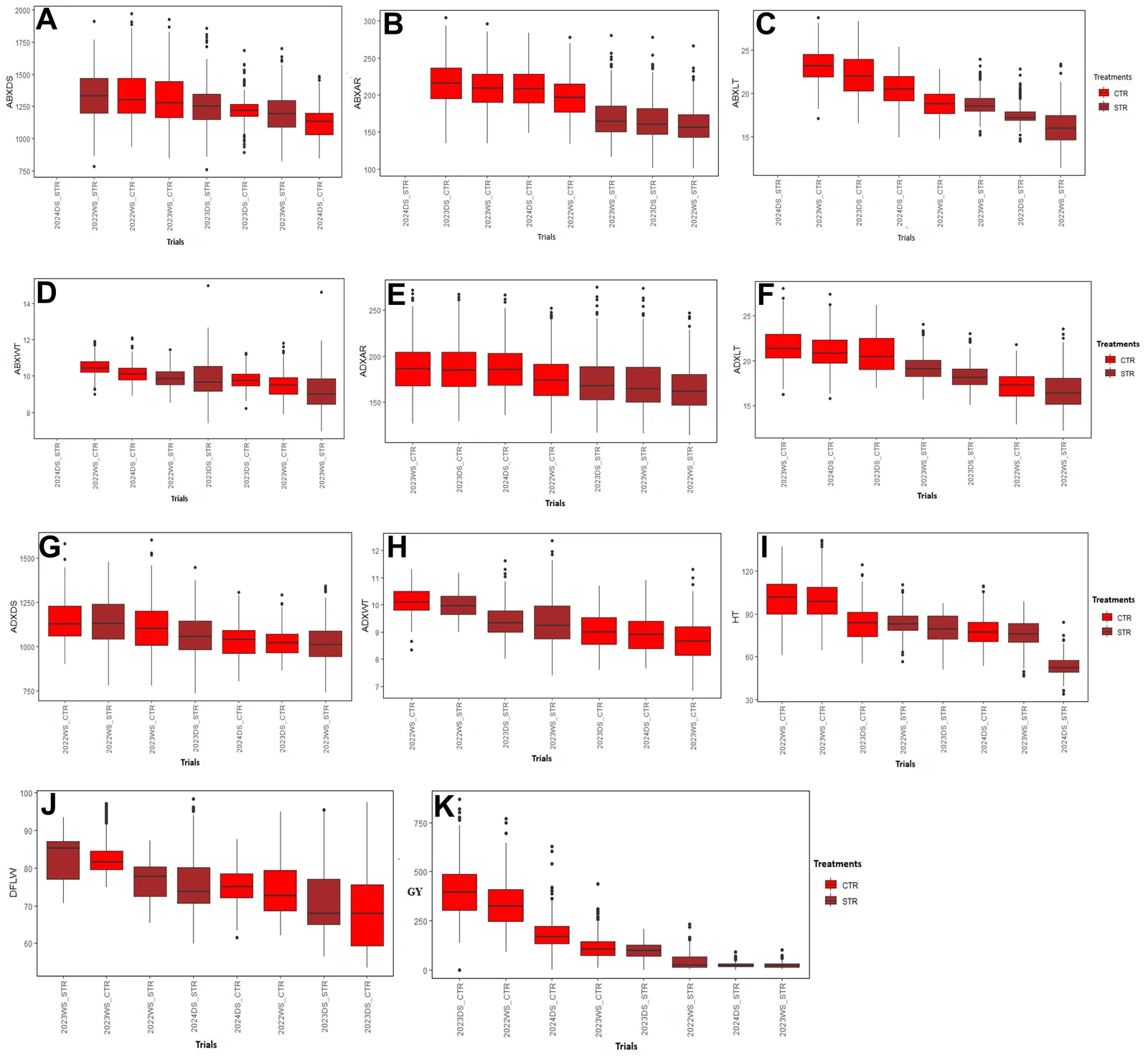
Box plots showing the distribution of agronomic and stomatal traits among 272 rice recombinant inbred lines evaluated under non-stress (CTR) and drought stress (STR) conditions across seasons. Boxes represent the interquartile range (IQR), horizontal lines indicate median values, whiskers extend to 1.5 × IQR, and points represent outliers. **(A)** abaxial stomatal density (ABXDS), **(B)** abaxial stomatal area (ABXAR), **(C)** abaxial stomatal length (ABXLT), **(D)** abaxial stomatal width (ABXWT), **(E) **adaxial stomatal area (ADXAR), **(F)** adaxial stomatal length (ADXLT), **(G)** adaxial stomatal density (ADXDS), **(H)** adaxial stomatal width (ADXWT), **(I)** plant height (HT), **(J)** days to flowering (DFLW) and **(K)** grain yield (GY).

Among agronomic traits, plant height declined under DS, with the strongest reduction in the final trial (**Figure 2I**). Days to flowering showed largely overlapping distributions between WW and DS, consistent with the weak genotype × condition interaction observed in the combined ANOVA (**Figure 2J**, **Table 2**). Grain yield showed the strongest drought response, with sharply reduced medians and narrower distributions under DS across seasons, although several high-yielding stress outliers indicated drought-adaptive RILs (**Figure 2K**). Overall, the boxplots confirm substantial genetic variation and trait-specific responses to drought stress within the RIL population.

**Table 2:** Phenotypic correlation between stomatal traits under non-stress and drought stress conditions.

Combined stress											
	ABXDS	ABXAR	ABXLT	ABXWT	ADXDS	ADXAR	ADXLT	ADXWT	DFLW	HT	GY
ABXDS	0.11	-0.39***	-0.40***	-0.31***	0.61***	-0.16*	-0.16*	-0.13	0.07	0.02	-0.08
ABXAR	-0.63***	0.07	0.92***	0.91***	-0.19**	0.34***	0.32***	0.24***	-0.01	-0.04	-0.09
ABXLT	-0.61***	0.93***	0.10	0.72***	-0.23***	0.31***	0.36***	0.21**	-0.12*	-0.04	-0.04
ABXWT	-0.49***	0.79***	0.56***	-0.01	-0.09	0.25***	0.21**	0.24***	0.01	0.00	-0.09
ADXDS	0.74***	-0.51***	-0.50***	-0.37***	0.03	-0.36***	-0.37***	-0.27***	0.06	-0.04	-0.02
ADXAR	-0.38***	0.49***	0.49***	0.31***	-0.46***	-0.05	0.94***	0.84***	0.03	-0.03	-0.03
ADXLT	-0.42***	0.45***	0.49***	0.23***	-0.46***	0.91***	-0.03	0.65***	-0.09	-0.01	0.02
ADXWT	-0.27***	0.42***	0.37***	0.36***	-0.38***	0.83***	0.57***	-0.10	0.04	-0.01	-0.02
DFLW	0.41***	-0.37***	-0.34***	-0.31***	0.40***	-0.31***	-0.31***	-0.24***	-0.03	-0.24***	-0.40***
HT	-0.03	0.01	-0.01	0.07	-0.04	0.03	0.02	0.04	0.08	0.1	0.43***
GY	0.03	-0.09	-0.09	-0.06	0.02	0.05	0.04	0.07	0.12	0.39***	-0.1
Combined well-watered											

Days to flowering , DFLW; plant height, HT; grain yield per square meter, GY; abaxial stomata density, ABXDS; abaxial stomatal area, ABXAR; abaxial stomatal length, ABXLT; abaxial stomatal width, ABXWT; adaxial stomatal density, ADXDS; adaxial stomatal area, ADXAR; adaxial stomatal length, ADXLT; and adaxial stomatal width, ADXWT. The correlation in stress and control is given and below the diagonal, respectively. Diagonal elements represent correlation between stress and control.

*, **,***Significant at p , 0.05, 0.01 and 0.001, respectively.

The broad-sense heritability estimates for the agronomic traits were high across seasons under both non-stress and stress conditions, indicating that a substantial proportion of the phenotypic variation was genetically controlled (**Supplementary Table 1a**). Under non-stress conditions, heritability for days to flowering ranged from 0.82 to 0.98, plant height from 0.91 to 0.98, and grain yield from 0.94 to 0.99 across the four seasons. Similarly, under drought stress conditions, heritability estimates remained high, ranging from 0.84 to 0.98 for days to flowering, 0.80 to 0.94 for plant height, and 0.95 to 0.97 for grain yield.

The combined analysis across seasons further confirmed the moderate to high heritability of the evaluated agronomic traits (**Table 1**). Under non-stress conditions, heritability values were 0.58 for DFLW, 0.75 for HT, and 0.54 for GY. Under drought-stress conditions, heritability increased slightly for days to flowering (0.66) but remained comparable for plant height (0.74), whereas grain yield showed a quite lower value (0.39). Compared to other traits, the heritability of GY was more affected by drought stress.

### 3.2 Stomatal traits performance under stress, non-stress conditions and across seasons

Striking differences were noted between the abaxial and adaxial leaf surfaces under both stress and non-stress conditions over seasons (**Supplementary Table 1b**). Under well-water conditions, IRRI154 exhibited seasonal variation in abaxial stomatal density, declining from 1,596.84 mm^-2^ (2022WS) to 1,274.80 mm^-2^ (2023DS), 1,442.26 mm^-2^ (2023WS), and 1,302.28 mm^-2^ (2024DS). Under drought, responses were season-dependent rather than unidirectional, ranging from 1,465.93 mm^-2^ (2022WS) to 1,411.72 mm^-2^ (2023DS) and 1,335.22 mm^-2^ (2023WS). In contrast, NERICA4 maintained relatively stable abaxial stomatal density, with well-water values of 980.00–1,068.61 mm^-2^ and stress values that were similar or slightly higher in 2022WS (1,351.28 vs. 1,068.61 mm^-2^) and 2023DS (1,070.68 vs. 1,049.02 mm^-2^). The progeny means abaxial density remained comparatively stable across environments (1,128.01 to 1,336.82 mm^-2^ under well-water and 1,202.23 to 1,337.86 mm^-2^ under stress), with the greatest reduction occurring in 2023WS (7.7%).

Adaxial stomatal density followed a similar trend but showed greater drought sensitivity. IRRI154 exhibited the largest stress-induced reduction in 2023WS, decreasing by 21.9% (1,519.56 to 1,187.00 mm^-2^), while a 13.1% reduction was observed in 2022WS (1,383.11 to 1,201.34 mm^-2^). Conversely, NERICA4 maintained comparable or slightly higher adaxial density under stress, suggesting a contrasting adaptive strategy. Progeny mean adaxial density changed only modestly across environments, with the largest decline (7.9%) occurring in 2023WS (1,107.78 to 1,020.05 mm^-2^), consistent with the abaxial response.

Conversely, phenotypic traits related to stomatal size were more responsive to water-limited condition. Both across parents and progeny, decreases in stomatal area and length were greater than similar shifts in density, especially on the abaxial surface, in response to drought stress. Under both seasons, IRRI 154 had a larger stomatal area, length and width compared to NERICA 4 on both leaf surfaces under well-watered conditions. In contrast, the drought stress reduced stomatal dimensions for both parents, and this effect was stronger in NERICA 4. Stomatal area, length and width in the abaxial surface of NERICA 4 were reduced from 214.78 to 171.82 μm^2^ (20.0%), 22.32 to 18.94 μm (15.1%) and 9.85 to 8.86 μm (10.0%), respectively. Likewise, on the adaxial surface also, stomatal area decreased from 194.42 to 160.20 μm^2^ (17.6%), stomatal length from 21.29 to 18.15 μm (14.7%) and stomatal width from 9.12 to 8.82 μm (3.3%), indicating that drought effects were more pronounced on stomatal dimensions than density.

A similar but more variable response was seen in the progeny for stomatal morphology in response to moisture regimes (**Table 1**), reflecting extensive segregation under contrasting moisture conditions. In RILs population, drought stress decreased stomatal traits on both leaf surfaces, although the magnitude differed among lines over the seasons. The progeny means also show that abaxial stomatal area decreased from 208.15 to 165.43 μm^2^ (20.5%), stomatal length decreased from 21.15 to 17.51 μm (17.2%), and stomatal width decreased from 10.04 to 9.68 μm (3.6%), respectively. On the adaxial surface, stomata decreased from 181.90 to 167.46 μm^2^ (7.9%), with a stomatal length from 20.07 to 17.91 μm (10.8%) and stomatal width from 9.11 to 8.95 μm (1.8%). We found significant drought-induced reductions in both stomatal area and stomatal length (26% and 34%, respectively) and that these were greater than changes in stomatal width (8% reduction for control), and greater on the abaxial surface, indicating that the lower epidermis is more sensitive to drought stress than the upper.

Broad-sense heritability estimates were high across seasons under both non-stress and drought-stress conditions (**Supplementary Tables 1a, b**), indicating a strong genetic contribution to the observed phenotypic variation. Under well-watered conditions, heritability estimates for stomatal density, area, and length were consistently high, often exceeding 0.80 and reaching values close to 0.99 in several seasons. Similarly, under drought stress, heritability remained high for most traits, particularly stomatal density, and area, suggesting that genetic effects remained stable despite differing environmental stress. The combined analysis across seasons and years (**Table 1**) confirmed these findings, with heritability values ranging from 0.61 to 0.83 under non-stress conditions and from 0.79 to 0.99 under drought stress. Remarkably, stomatal area traits exhibited the highest heritability estimates under stress, whereas stomatal width showed comparatively lower values. The consistently high heritability observed in both seasons and combined analyses indicate that stomatal density and size traits are predominantly under genetic control and can be effectively exploited in breeding programs aimed at improving drought adaptation in rice.

### 3.3 Phenotypic correlation between agronomic and stomatal traits under non-stress and drought stress conditions

Phenotypic correlations among stomatal and agronomic traits under drought stress and well-watered conditions differed (**Table 2**). Under well-watered conditions, corresponding stomatal traits deploy strong inter-surface positive correlation coefficients on the abaxial and adaxial leaf surfaces (**Table 2**). A strong positive correlation was found between abaxial stomatal density (ABXDS) and adaxial stomatal density (ADXDS; r , 0.74, P< 0.001), and between abaxial and adaxial stomatal area (ABXAR-ADXAR), (r , 0.49, P< 0.001), stomatal length (ABXLT-ADXLT), (r , 0.49, P< 0.001) and stomatal width (ABXWT-ADXWT), (r , 0.36, P< 0.001). Stomatal size traits within each leaf surface were highly correlated. On the abaxial surface, we found strong relationships with stomatal area and stomatal length (r , 0.93, P< 0.001) and width (r , 0.79, P< 0.001); on the adaxial surface, stomatal area was similarly and strongly related with both stomatal length (r , 0.91, P< 0.001) and width (r , 0.83, P< 0.001). Conversely, on both leaf surfaces, stomatal size traits were negatively correlated with stomatal density, especially between ABXDS and ABXAR (r , −0.63, P< 0.001) and between ADXDS and ADXAR (r , −0.46, P< 0.001).

Under drought stress conditions, similar correlation patterns were observed among stomatal traits. Strong positive associations occurred between corresponding adaxial and abaxial stomatal traits, while stomatal size traits remained highly correlated within each leaf surface. Abaxial stomatal area showed strong positive relationships with stomatal length (r , 0.92, P< 0.001) and width (r , 0.91, P< 0.001), whereas adaxial stomatal area was strongly associated with stomatal length (r , 0.94, P< 0.001) and width (r , 0.84, P< 0.001). Stomatal density remained negatively associated with stomatal size traits under drought stress, particularly between ABXDS and ABXAR (r , -0.39, P< 0.001), ABXLT (r , -0.40, P< 0.001), ADXAR (r , -0.16, P< 0.05), and ADXLT (r , -0.16, P< 0.05). Likewise, ADXDS exhibited negative correlations with both adaxial and abaxial stomatal size traits. These results indicate that drought stress maintained the contrary relationship between stomatal density and dimensions observed under non-stress conditions.

An interesting and biologically important finding was the near-complete absence of significant correlations between the same stomatal and agronomic traits measured under non-stress and drought stress conditions, across all traits examined (**Table 2**). Specifically, abaxial stomatal density under non-stress showed no significant correlation with abaxial stomatal density under stress (r , 0.11, P > 0.05), and the same pattern held for abaxial area (r , 0.07, ns), abaxial length (r , 0.10, ns), and abaxial width (r , -0.01, ns). Similarly, adaxial stomatal density (r , 0.03, ns), adaxial area (r , -0.05, ns), adaxial length (r , -0.03, ns), and adaxial width (r , -0.10, ns) showed no significant within-trait, cross-environment correlations. The agronomic traits followed the same pattern: days to flowering (r , -0.03, ns), plant height (r , 0.10, ns), and grain yield (r , -0.10, ns) were not significantly correlated between non-stress and stress conditions. These results indicate that the rank ordering of genotypes for all measured traits was different between non-stress and drought stress environments, reflecting strong genotype × environment interaction for both stomatal traits and agronomic traits. The absence of significant within-trait across season-water regimes correlations underscores the importance of evaluating genotypes under both conditions independently.

Relationships between stomatal and agronomic traits were weak to moderate but revealed several important patterns. Days to flowering under non-stress condition was significant and negatively correlated with grain yield under stress (r , -0.40, P< 0.001), indicating that later-flowering genotypes produced lower grain yields under drought. Days to flowering was also positively correlated with stomatal density on both surfaces under stress abaxial (r , 0.41, P< 0.001), adaxial (r , 0.40, P< 0.001) and negatively correlated with stomatal area (abaxial; r , -0.37, P< 0.001; length r , -0.34, P< 0.001) under stress, suggesting that later-flowering genotypes generally have higher stomatal density and smaller stomatal area under drought. Additionally, days to flowering under non-stress was negatively correlated with plant height under stress (r , -0.24, P< 0.001), further linking phenological timing to overall plant performance. Plant height under non-stress was positively correlated with grain yield under stress (r , 0.43, P< 0.001), indicating that taller genotypes maintained greater yield capacity under water deficit, reflecting superior biomass accumulation and assimilate partitioning. In contrast, all stomatal traits under non-stress showed no significant correlations with grain yield under stress (r , -0.02 to -0.09, all ns). Thus, indicating that non-stress stomatal morphology alone does not predict drought-environment agronomic performance, and that integration of stomatal traits with phenological and morphological indicators may be necessary for effective indirect selection. Under drought-stress conditions, grain yield (g m^-2^) (GY) showed weak associations with most stomatal traits measured under the same stress conditions (**Table 2**). None of the stomatal density traits, including ABXDS (r , -0.08) and ADXDS (r , -0.02), were significantly correlated with grain yield under stress. Similarly, stomatal size traits, including ABXAR (r , -0.09), ABXLT (r , -0.04), ABXWT (r , -0.09), ADXAR (r , -0.03), ADXLT (r , 0.02), and ADXWT (r , -0.02), exhibited non-significant relationships with grain yield. These results indicate that variation in stomatal density and size under drought stress did not directly contribute to differences in grain yield among the RILs.

### 3.4 Correlation of traits performance across environments

Trait stability across seasons and water regimes was assessed using environment-wise correlation matrices (**Figure 3**). The figures revealed distinct patterns among agronomic and stomatal traits under well-watered and drought stress conditions, reflecting differential environmental influences on trait expression. Among stomatal traits, abaxial stomatal area (ABXAR) and length (ABXLT) exhibited strong positive correlations across environments, particularly between 2022WS_STR and 2023WS_STR, indicating high stability under drought stress. Abaxial stomatal density (ABXDS) showed a similar pattern, whereas abaxial stomatal width (ABXWT) displayed weaker, more variable correlations, indicating greater phenotypic plasticity.

**Figure 3:**
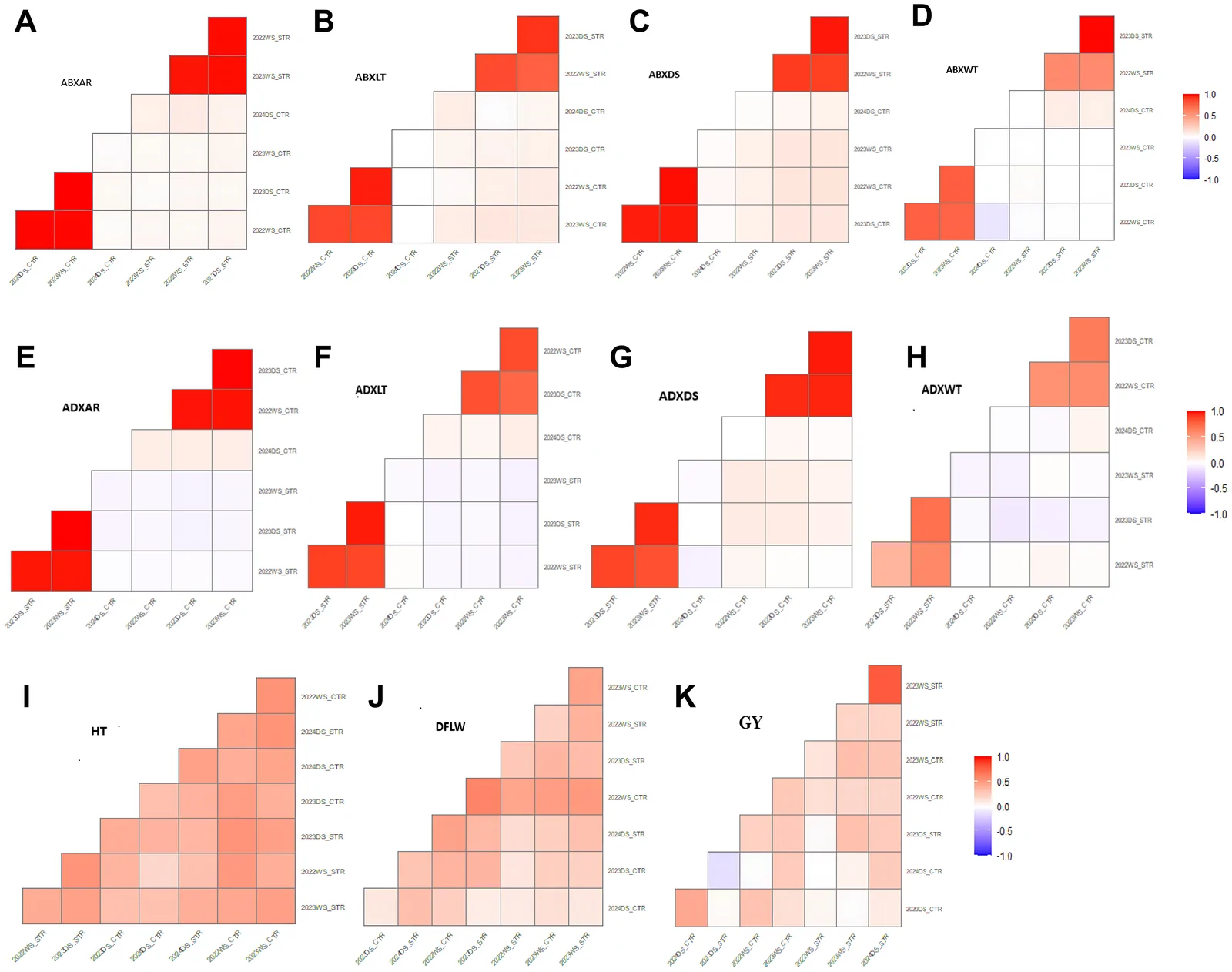
Pairwise correlation coefficients among the environments for **(A)** abaxial stomatal area (ABXAR), **(B)** abaxial stomatal length (ABXLT), **(C)** abaxial stomatal density (ABXDS), **(D)** abaxial stomatal width (ABXWT), **(E)**adaxial stomatal area (ADXAR), **(F)** adaxial stomatal length (ADXLT), **(G)** adaxial stomatal density (ADXDS), **(H)** adaxial stomatal width (ADXWT), **(I)** plant height (HT), **(J)** days to flowering (DFLW) AND **(K)** grain yield (GY) measured in the RILs. This illustrates association between environments under drought stress (STR) and well-watered/control (CTR) conditions during the 2022-2024 growing seasons. Red colors indicate strong positive correlations, white indicates weak correlations, and blue indicates negative correlations.

Adaxial stomatal traits showed stronger environmental reliability. Adaxial stomatal area (ADXAR) and length (ADXLT) exhibited high positive correlations among control environments, while adaxial stomatal density (ADXDS) showed moderate-to-strong consistency across both moisture regimes. Adaxial stomatal width (ADXWT) similarly displayed weaker correlations and near-zero or slightly negative relationships, indicating increased environmental sensitivity.

Among agronomic traits, DFLW demonstrated the highest stability, with consistently positive correlations across all environments indicating that genotype ranking for days to flowering remained unchanged across seasons. Plant height (HT) showed moderate positive correlations across environments, reflecting limited but measurable environmental influence. Grain yield (GY) exhibited the most variable correlation pattern, with weak and occasionally negative correlations between stress and non-stress environments, confirming strong genotype x environment interaction and crossover responses reflecting the complexity of drought adaptation.

Days to flowering, plant height, and stomatal size traits showed relatively high environmental stability, whereas grain yield and stomatal width exhibited greater sensitivity, underscoring the necessity of multi-environment evaluation to identify consistent drought-adaptive genotypes.

### 3.5 Combined analysis of variance for agronomic and stomatal traits across the seasons and treatments

The combined ANOVA revealed highly significant genotypic variation for all agronomic and stomatal traits (**Table 3**). Genotype mean squares ranged from 1.19 for ADXWT to 111,954.0 for grain yield (GY), indicating substantial genetic variation among the RILs. Particularly large genotypic effects were observed for grain yield (111,954.0, P< 0.001), adaxial stomatal density (26,243.0, P<0.001), abaxial stomatal density (25,873.0, P< 0.001), plant height (100.1, P< 0.001), and days to flowering (22.2, P< 0.001).

**Table 3:** Analysis of variance for agronomic and stomatal traits across the seasons and treatments.

Source of variation	DF	ABXDS	ABXAR	ABXLT	ABXWT	ADXDS	ADXAR	ADXLT	ADXWT	DFLW	HT	GY
Genotype	277	25873.0***	83.7***	7.1***	1.6***	26243.0***	64.2***	7.9***	1.19***	22.2***	100.1***	111954.0***
Condition	1	521.0	3479.0**	48.9*	1.0	588.0	1012.4*	16.10*	0.8	67.3*	245.2*	842490.0*
Genotype : Condition	277	112251.0***	3688.3***	5.9***	1.8***	76034.0***	2616.6***	8.4***	1.44***	5.4	24.6***	92773.0***

Note: condition (nested seasons x water regime); Days to flowering , DFLW; plant height, HT; grain yield , GY; abaxial stomata density, ABXDS; abaxial stomatal area, ABXAR; abaxial stomatal length, ABXLT; abaxial stomatal width, ABXWT; adaxial stomatal density, ADXDS; adaxial stomatal area, ADXAR; adaxial stomatal length, ADXLT; and adaxial stomatal width, ADXWT, *, **, *** Significant at the 0.05, 0.01, 0.001 probability level respectively.

The nested condition effect (season × water regime) significantly influenced several traits. The largest condition mean squares were recorded for grain yield (842,490.0, P< 0.05), abaxial stomatal area (3,479.0, P< 0.01), adaxial stomatal area (1,012.4, P< 0.05), adaxial stomatal density (588.0), abaxial stomatal density (521.0), plant height (245.2, P< 0.05), and days to flowering (67.3, P< 0.05). In contrast, stomatal width traits (ABXWT and ADXWT) showed comparatively small condition effects.

Genotype × condition interactions were highly significant for most stomatal traits and grain yield, indicating differential genotype responses across seasons and water regimes. Interaction mean squares were particularly high for ABXDS (112,251.0, P< 0.001), GY (92,773.0, P< 0.001), ADXDS (76,034.0, P< 0.001), ABXAR (3,688.3, P< 0.001), and ADXAR (2,616.6, P< 0.001). Significant interactions were also detected for stomatal length and width traits on both leaf surfaces. However, the interaction effect for days to flowering was not significant, suggesting stable flowering responses across environments.

The magnitude of the genotype and genotype × condition mean squares for grain yield and stomatal traits demonstrates substantial genetic variability and environmental responsiveness within the population, providing a strong basis for identifying drought-adaptive genotypes and associated stomatal characteristics.

### 3.6 Genotype × environment interaction for agronomic and stomatal traits

Across all evaluated agronomic and stomata traits, the first two principal components (PC1 and PC2) of GGE biplots explained a substantial proportion of the genotype plus genotype × environment (G + GE) variation, accounting for approximately 85% of the total variation (PC1 ≈ 50%, and PC2 ≈ 35%) (**Figure 4**). The high cumulative variation indicates that the biplots adequately represent the interaction and stability patterns among genotypes across seasons and water regimes. The angles between water regimes vectors further revealed the relationships among test environments. Acute angles (<30°) were consistently observed within the same moisture regime, indicating strong positive correlations and consistent genotype performance rankings. Conversely, obtuse angles (120° - 180°) predominated between drought-stress (STR) and well-watered control (CTR) environments, reflecting strong crossover interactions and contrasting genotype performance rankings across moisture regimes. Environments with longer vectors exhibited greater discriminating ability for genotype evaluation, whereas shorter vectors were less effective in differentiating genotypic performance.

**Figure 4:**
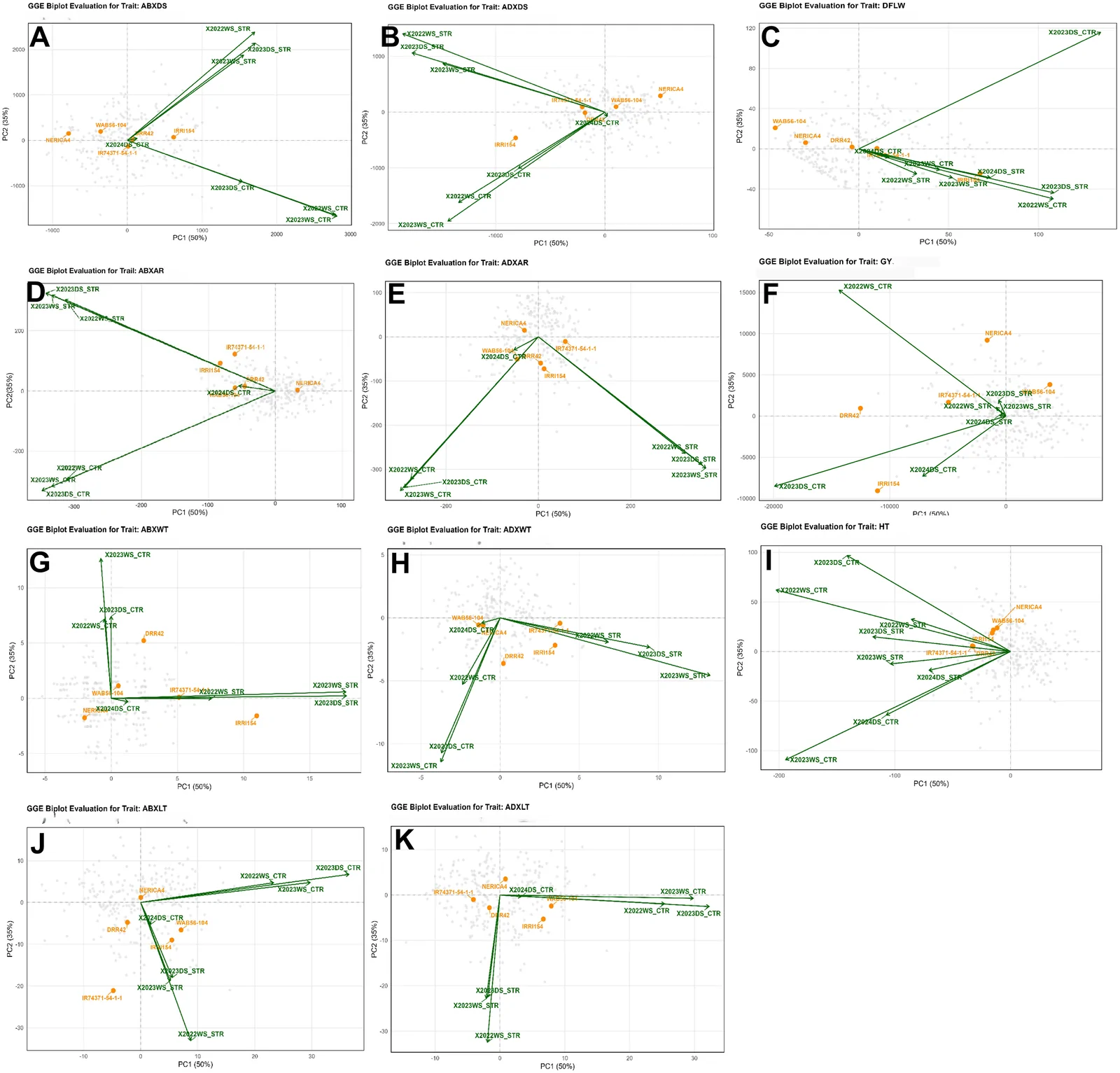
GGE biplot analysis of stomatal, agronomic, and grain yield traits showing genotype performance and genotype × environment interactions across control and drought-stress conditions in a rice recombinant inbred line population. **(A)** abaxial stomatal density (ABXDS), **(B)** adaxial stomatal density (ADXDS), **(C)** days to flowering (DFLW), **(D)** abaxial stomatal area (ABXAR), **(E)** adaxial stomatal area (ADXAR), **(F)** grain yield (GY), **(G)** abaxial stomatal width (ABXWT), **(H)** adaxial stomatal width (ADXWT), **(I)** plant height (HT), **(J)** abaxial stomatal length (ABXLT), and **(K)** adaxial stomatal length (ADXLT) measured in the RILs.

Drought- stress environments exhibited longer vectors than control environments, demonstrating superior discriminating power for yield evaluation. IRRI154 was positioned closer to stress-associated sectors, exhibiting higher yield stability under drought, whereas NERICA 4 was more strongly associated with control environments. Several RILs occupied the vertex positions and emerged as leading genotypes for grain yield in specific environments.

Most environments exhibited acute angles (<30°) for days to flowering, indicating positive correlations for flowering responses across seasons. However, 2023DS_CTR formed a wide angle (> 90°) with most other environments and displayed the longest vector, indicating both a distinct environmental response pattern and high discriminating ability. IRRI154 was positioned toward the sectors associated with delayed flowering under stress, whereas NERICA4 was positioned in the opposite direction, reflecting earlier flowering trends. Vertex RILs were identified as winning genotypes for specific environmental sectors.

IRRI 154 was positioned toward the positive PC1 direction associated with greater plant height under stress, whereas NERICA 4 occupied a more central position, indicating moderate environmental responsiveness. Several RILs constituted winning genotypes across different sectors.

Stomatal density for both abaxial and adaxial (ABXDS & ADXDS), exhibits acute angles (< 25°) indicating strong environmental consistency within the same moisture regime, whereas obtuse angles (130° -- 170°) between stress and control environments suggest contrasting genotype responses. The longest vectors were observed for 2023DS_STR and 2023DS_CTR, indicating superior discriminating power. IRRI 154 was positioned toward the stress-environment sector and was associated with higher stomatal density under drought conditions, whereas NERICA 4 was located opposite the stress vectors, reflecting lower stomatal density under stress. Several advanced RILs occupied the vertex positions and represented winning genotypes within their respective sectors.

Stomatal area (ABXAR & ADXAR) shows that stress environments clustered tightly (< 20°) while showing strong negative associations (140° -- 180°) with control environments. The 2023DS_STR and 2023WS_STR environments displayed the greatest discrimination among genotypes. IRRI154 and several elite RILs were associated with larger stomatal area under drought stress, whereas NERICA4 was positioned near the average environment coordinate (AEC), indicating stable performance across environments.

However, stomatal dimensions (ABXWT, ABXLT, & ADXLT) displayed acute angles among stress environments (<20°) indicated high-performance consistency for stomatal width and length under drought. 2023DS_STR consistently displayed the greatest vector length and discriminating power. IRRI154 was associated with longer and wider stomata under stress-environments, while NERICA4 aligned closely with control environments. The winning genotypes differed among sectors, indicating substantial crossover interaction.

The GGE biplot analysis effectively partitioned the genotype × environment interaction, identifying 2023DS_STR as the most discriminating test environment and establishing patterns of genotypic performance stability across moisture regimes. While these biplots effectively delineate trait stability and environmental discrimination, functional inferences regarding drought adaptation mechanisms are evaluated through direct integration with yield correlations and physiological index analyses.

### 3.7 Principal component analysis of stomatal and agronomic trait variation among rice recombinant inbred lines under well-watered and drought stress conditions.

Principal component analysis (PCA) of stomatal and agronomic traits under well-watered conditions revealed distinct patterns of trait association and genotypic variation within the RIL population (**Figure 5**). The first two principal components dimension1 and 2 (Dim1 and Dim2) collectively explained 55.5% of the total phenotypic variation, with Dim1 accounting for 42.4% and Dim2 accounting for 13.1%, indicating that these two axes adequately captured the major sources of trait variation among genotypes.

**Figure 5:**
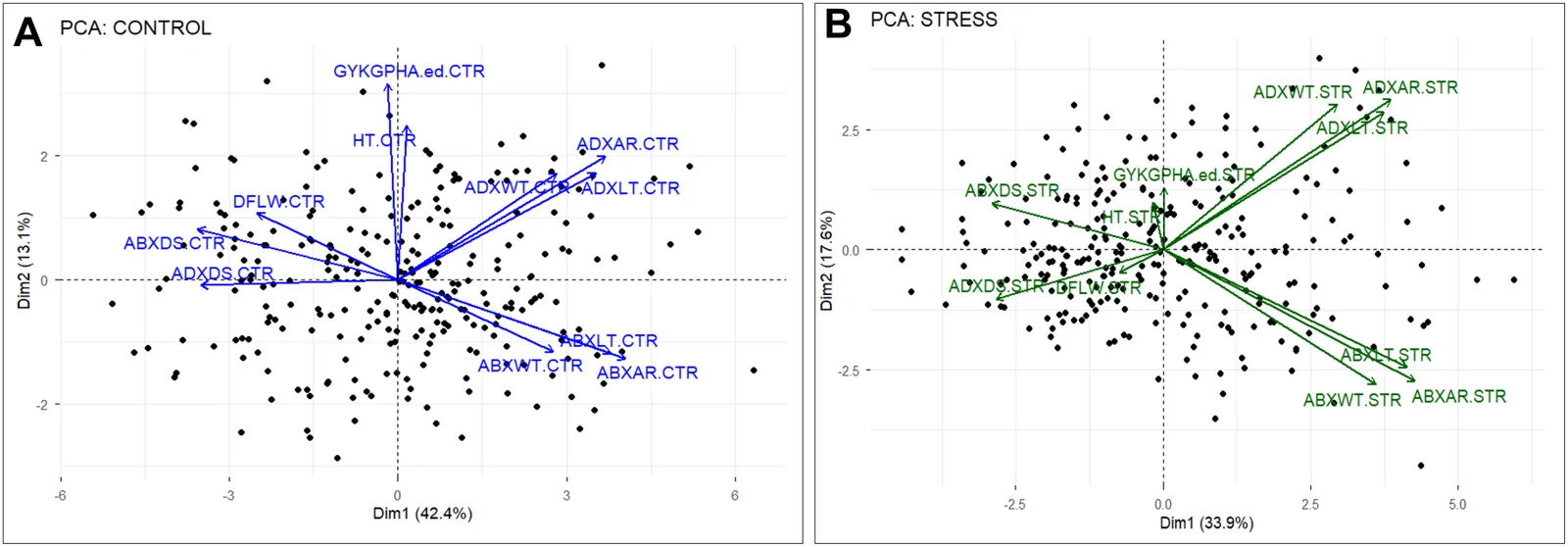
Principal component analysis (PCA) biplot illustrating phenotypic relationships among adaxial and abaxial stomatal density (ADXDS, ABXDS), stomatal area (ADXAR, ABXAR), stomatal length (ADXLT, ABXLT), stomatal width (ADXWT, ABXWT), plant height (HT), days to flowering (DFLW), and grain yield (GY) in 272 rice recombinant inbred lines under non-stress and stress conditions. PC1 and PC2 under well-watered **(A)** explained 42.4% and 13.1% of the total phenotypic variation, respectively, accounting for 55.5% of the total variation while under stress **(B)** 33.9% and 17.6% of the total phenotypic variation, respectively, accounting for 51.5% of the total variation. Trait vectors indicate the magnitude and direction of trait contributions, while the distribution of genotypes reflects underlying phenotypic diversity. within the population.

Along Dim1, stomatal size traits on both leaf surfaces were the primary contributors, with abaxial stomatal area (ABXAR), abaxial stomatal length (ABXLT), and abaxial stomatal width (ABXWT) loading strongly in the positive direction, alongside their adaxial counterparts (ADXAR, ADXLT, ADXWT). The close alignment of abaxial and adaxial stomatal size vectors indicates strong positive associations among stomatal dimensions across both leaf surfaces, suggesting coordinated developmental regulation of stomatal morphology under non-stress conditions. Stomatal density traits, however, loaded strongly in the negative direction along Dim1, with abaxial stomatal density (ABXDS) and adaxial stomatal density (ADXDS) exhibiting vectors opposing those of stomatal size traits, reflecting an inverse relationship between stomatal number and stomatal dimensions.

Along Dim2, agronomic traits were the primary drivers of variation. Grain yield and plant height loaded positively, while days to flowering (DFLW) loaded in the negative direction, suggesting that taller, higher-yielding genotypes tended to flower later under non-stress conditions. The near-orthogonal orientation of agronomic trait vectors relative to stomatal size trait vectors along Dim1 indicates limited direct association between agronomic performance and stomatal morphology under well-watered conditions.

PCA of stomatal and agronomic traits under drought stress revealed broadly similar but quantitatively distinct patterns compared to well-watered conditions (**Figure 5**). Dim1 and Dim2 collectively explained 51.5% of total phenotypic variation, with Dim1 accounting for 33.9% and Dim2 for 17.6%. The reduction in total variation explained by Dim1 relative to the control PCA (42.4%) suggests that drought stress redistributed phenotypic variation more evenly across multiple axes, reflecting increased complexity of trait relationships under water-deficit conditions.

Along Dim1, stomatal size traits again dominated, with ABXAR, ABXLT, and ABXWT loading strongly in the positive direction in the lower right quadrant, while ADXAR, ADXLT, and ADXWT loaded positively in the upper right quadrant. Particularly, abaxial and adaxial stomatal size vectors were more angularly separated under stress than under control conditions, suggesting that drought induced surface-specific divergence in stomatal morphological responses. Stomatal density traits loaded strongly in the negative direction, with ABXDS and ADXDS density vectors opposing stomatal size traits along Dim1, maintaining the inverse density-size relationship observed under non-stress conditions.

Along Dim2, grain yield and plant height (HT) loaded positively, while DFLW loaded negatively, consistent with the control PCA. The broad scatter of genotypic scores confirmed substantial phenotypic variation among RILs under drought stress, supporting the potential for identifying drought-adaptive genotypes.

The wide dispersion of genotypic scores across the biplot confirmed the broad phenotypic variation present within the RIL population for both stomatal and agronomic traits, providing a strong basis for selection of drought-adaptive phenotypes.

## 4 DISCUSSION

Stomata traits, particularly density, are known to be important for drought tolerance in rice. However, there is not much information available that would enable breeders to use these traits as a selection criterion in breeding for drought tolerance. We studied the *GxE* of stomatal traits in a recombinant inbred population of rice and also explored important parameters such as genetic variation, reliability of selection, stability of the trait, and correlation with grain yield.

### 4.1 Phenotypic diversity, transgressive segregation, and single-trial repeatability

The extensive phenotypic variation observed for all four stomatal traits (stomatal density, area, length, and width) on both the abaxial and adaxial leaf surfaces confirms a highly polymorphic genetic architecture within this recombinant inbred line (RIL) population. The broad distributions and more than twofold variation among the progeny lines for both density and individual dimensions align with natural variation ranges reported in cultivated and wild *Oryza* species (Ohsumi et al., 2007; Chatterjee et al., 2020). This structural variation confirms that the genetic components regulating both the numerical frequency and spatial geometry of stomata are highly polymorphic within this germplasm, providing a robust baseline for breeding selection.

A key feature of this population is the widespread presence of transgressive segregation across all examined stomatal density and size traits on both leaf surfaces (**Figure 2**). Remarkably, the upper limits of progeny abaxial stomatal density substantially exceeded maximum values typically reported in cultivated rice literature (**Table 1**). Concurrently, substantial transgressive segregation was observed for individual stomatal size traits under both moisture regimes (**Table 1**). This bidirectional transgressive variation was evident on both leaf surfaces, producing novel progeny phenotypes with guard cell areas, lengths, and widths that extended well beyond the boundaries of both parental lines. Recombination between the distinct component genetic backgrounds evidently generated novel allelic combinations that are under-represented in conventional germplasm collections. While individual lines displayed unique configurations where higher density occasionally coupled with larger dimensions at the absolute line level, a robust inverse density-size trade-off was clearly expressed across the segregating progeny population as a whole.

Within individual seasonal trials, broad-sense heritability estimates, which functionally reflect phenotypic repeatability, frequently exceeded 0.80 and reached values close to 0.99 for both density and individual size traits under both moisture treatments. This high repeatability validates the micro-imprint extraction and imaging pipeline as a highly reliable, low-error protocol for capturing true genetic variation within controlled environments, confirming that both numerical and dimensional epidermal structures are highly exploitable target traits for genetic selection across RIL populations.

### 4.2 Spatial coordination and surface asymmetry of stomatal traits

The comparison between the abaxial and adaxial leaf surfaces reveals a distinct pattern in which both surfaces shared highly coordinated genetic relationships yet remain structurally and functionally dissimilar. On one hand, strong positive inter-surface correlation coefficients, particularly between abaxial and adaxial stomatal density and stomatal area, demonstrate a highly synchronized developmental program (**Table 2**). This tight correlation suggests that the broad genetic mechanisms regulating epidermal patterning, cell differentiation, and stomatal spacing operate symmetrically across the entire flag leaf blade.

On the other hand, the two surfaces display marked numerical and operational dissimilarity. Under well-watered conditions, abaxial stomatal density was consistently higher than adaxial density across seasons (**Table 1**), confirming that the lower epidermis carries the bulk of the plant’s gas exchange capacity (Willmer and Fricker, 1996; Bertolino et al., 2019; Wall et al., 2022). Furthermore, the two surfaces responded with highly unequal sensitivity to environmental stress (**Figure 2**). The abaxial epidermis exhibited significantly higher environmental sensitivity and structural flexibility, whereas the adaxial surface remained far more rigid across treatments. This localized abaxial flexibility allows the lower leaf surface to act as the primary active barrier against excessive transpirational water loss during severe water deficits.

### 4.3 Physiological and morphological plasticity under drought stress

Drought stress exerted a differential morpho-developmental impact across traits, primarily modifying stomatal dimensions rather than stomatal density (**Table 3**). Across surfaces and seasons, changes in stomatal density were modest and variable, fluctuating between minor increases and localized reductions (**Table 1**). This indicates that, after epidermal cells are initiated, numerical stomatal density remains relatively stable under fluctuating water regimes.

Conversely, stomatal size traits responded robustly and uniformly to drought stress, driving severe structural shifts across the population bounds (**Figure 2**; **Table 1**). In the segregating RIL population, reproductive-stage water deficit induced a coordinated downsizing of guard cell dimensions, which was prominently driven by sharp decreases in stomatal area and length rather than width. This dimensional compression was markedly more pronounced on the abaxial epidermis, whereas the adaxial surface exhibited a more rigid, structurally stable response across treatments. The formation of smaller, shorter stomatal dimensions under water deficit is a documented adaptive response in cereals. Shorter guard cells facilitate significantly more rapid stomatal closing kinetics, allowing the plant to prevent catastrophic hydraulic failure during sudden stress intervals while maximizing water-use efficiency (WUE) during transient water supply (Franks and Farquhar, 2006; Caine et al., 2018; Pitaloka et al., 2022).

### 4.4 Multi-environment stability dynamics, g×e crossover interactions, and combined heritability

Evaluating trait stability across fluctuating seasonal environments and moisture regimes is crucial for determining the utility of stomatal features in crop breeding. Analysis of variance (ANOVA) revealed highly significant genotype × condition interactions (P< 0.001) for stomatal density and all six stomatal size traits (**Table 2**), indicating strong phenotypic plasticity and crossover rank shifts between well-watered and reproductive-stage drought conditions (**Figure 3**). However, environment-wise correlation matrices and GGE biplot analyses showed that these traits are highly stable within specific production systems (**Figures 3**, **4**). Acute vector angles predominated among test environments within the same moisture regime, demonstrating consistent genotype rankings across seasons (**Figure 4**). Abaxial stomatal density, area, and length were most stable under drought stress across years, indicating that the genetic mechanisms regulating stress-induced adjustments are reliable and predictable (**Figure 3**).

This stability is further supported by contrasting single-trial heritability with the combined multi-environment analysis. High broad-sense heritability estimates from individual trials are not uncommon in rice under either drought or irrigated conditions and have been repeatedly reported (Venuprasad et al., 2009; Shaibu et al., 2018; Venkateshwarlu et al., 2022); our results are consistent with these reports. However, because single-environment heritability partitions only genotypic and residual variances, it absorbs G × E interaction variance into the genetic component and inflates individual estimates. When G × E variance was explicitly separated across seasons, complex agronomic traits declined sharply, with grain yield heritability dropping substantially under drought stress (**Table 1**).

Environmental influences on stomatal traits have been reported previously in rice and other crops (Caine et al., 2018; Wall et al., 2022; Hõrak, 2026), but most prior studies examined few traits under single or controlled conditions, limiting their applicability to field-based breeding. The present study extends this knowledge by quantifying G × E interactions for multiple density and size traits on both leaf surfaces across four seasons under contrasting water regimes in a large RIL population.

In contrast to grain yield, stomatal traits maintained robust combined heritability across environments. Combined heritability for abaxial stomatal density and area remained high, reaching exceptional values under drought stress, and stomatal length also showed strong heritability, while width was lower, confirming it as a more plastic dimension (**Table 1**). These findings indicate that stomatal density and size traits, particularly area and length, retain stable genetic control across contrasting treatments. Their capacity to outperform the heritability of grain yield under stress suggests that these anatomical traits provide stable, unmasked genetic signals, making them strong target traits for multi-environment breeding pipelines.

### 4.5 Genotypic interrelationships, physiological buffering, and breeding implications for yield improvement

Analyzing correlations between stomatal morphology and agronomic performance is essential for defining selection strategies in drought-resilience breeding. The phenotypic correlation matrix (**Table 2**) showed that stomatal traits measured under drought stress exhibit weak direct correlations with grain yield under the same stress conditions, with abaxial stomatal density and abaxial stomatal area indicating near-zero linear relationships. Rather than indicating a lack of functional utility, this uncoupled relationship suggests that stomatal traits operate as upstream components of stress resilience that do not translate directly into linear changes in sink capacity.

This indirect mode of action is further supported by other agronomic correlations in the matrix; for example, plant height under control conditions positively correlated with grain yield under stress, while days to flowering under control conditions was significantly negatively correlated with yield under stress, confirming that vegetative biomass and phenological timing are major concurrent factors associated with yield performance under water deficit (**Table 2**).

Because these relationships are based on static phenotypic and genetic correlations within a single mapping population, formal selection response studies using divergent stomatal architectures are needed to validate their direct effects on yield under drought. Specifically, future breeding research must actively select for divergent stomatal configurations (e.g., establishing high vs. low density or large vs. small area sub-populations) and evaluate the resulting impact on grain yield under controlled reproductive-stage stress. Until these dynamic selection-response studies are executed on derived lines, stomatal attributes should be treated as secondary traits rather than standalone selection criteria. Importantly, direct selection for grain yield under managed drought stress environments remains highly effective for generating stable genetic gains, as firmly established by Venuprasad et al. (2007), Venuprasad et al, 2008). However, because raw grain yield under stress can still be subject to environmental masking across seasons, integrating highly heritable secondary traits, such as abaxial stomatal density and area, can significantly strengthen selection efficiency and accelerate breeding cycles in multi-environment trials (**Table 1**).

Based on these parameters, we suggest that future breeding programs and large-scale germplasm screenings can pragmatically focus on abaxial stomatal density under well-watered control conditions alone as a unified proxy. This operational approach allows breeders to completely bypass the labor-intensive tracking of individual stomatal size traits and eliminate the need for stress-plot epidermal phenotyping, supported by several compelling biological and practical advantages. First, this approach avoids tissue distortion: drought stress triggers severe leaf rolling and tissue shrinkage that distorts the epidermal layout, ruining slide impressions and introducing measurement errors, whereas screening under control conditions ensures flat, reliable imprints. Second, this approach captures constitutive variation, as principal component analysis demonstrates that screening under optimal, non-stress conditions successfully captures the primary genetic variation governing overall epidermal development (**Figure 5**). Third, persistent structural trade-offs are preserved, since control-plot stomatal density maintains a robust inverse relationship with stomatal area and length (**Table 2**), allowing selection for density to predictably drive size adaptations without measuring guard cell dimensions (**Figure 5**). Fourth, this approach benefits from high heritability, as stomatal density under control conditions preserves excellent genetic control (**Table 1**), ensuring predictable genetic gains across segregating generations. Finally, this approach offers phenotyping efficiency, since counting stomatal frequency per unit area on clean control leaves is fundamentally faster, simpler, and less error-prone than tracing individual guard cell dimensions on deformed stress leaves.

By treating control-plot abaxial density as a unified, highly heritable target trait, breeding programs can integrate this anatomical signature with phenological indicators (like days to flowering) and primary yield components in a multi-trait index. This operational shortcut eliminates the primary constraints both manual labor bottlenecks and stress-induced tissue distortions that currently hinder the deployment of epidermal traits, allowing breeders to rapidly screen expansive genebank collections and large-scale segregating populations to make the development of climate-resilient, water-efficient rice cultivars an operationally viable reality.

### 4.6 Accelerating selection pipelines via deep learning and automation

Although focusing on stomatal density alone under control conditions dramatically improves screening efficiency, manual characterization remains a major operational bottleneck. In this study, the manual processing of thousands of microscopic images and individual stomata highlights the critical labor constraints that have historically blocked the deployment of anatomical traits in routine, large-scale breeding programs. To fully exploit stomatal density as a unified target across thousands of breeding lines, researchers can leverage recent advances in artificial intelligence, computer vision, and deep learning. Specialized convolutional neural network (CNN) pipelines and automated segmentation software including StomataCounter, DeepStomata, and StomataScorer offer a powerful pathway to accelerate this process (Fetter et al., 2019; Wolny et al., 2020; Gibbs et al., 2021). Utilizing models built on optimized architectures (such as Mask R-CNN or U-Net), these platforms can automate object detection and rapidly calculate stomatal numbers per unit leaf area with high accuracy and low observer bias.

By integrating automated image-processing tools into field pipelines, breeders can easily overcome the classical phenotyping bottleneck. This deep-learning framework allows for the rapid processing of extensive genebank collections, expansive mapping families, and large-scale segregating populations within timeframes that match standard breeding cycles. Combining AI-driven stomatal counting with genomic selection and marker-assisted breeding will ultimately turn the optimization of stomatal density from a labor-intensive theoretical concept into a scalable, high-throughput reality for modern climate-smart crop development.

## 5 Conclusion

This study establishes a comprehensive multi-environment evaluation of stomatal architecture in a rice RIL population. While reproductive-stage drought stress severely compromised grain yield and plant height, stomatal traits exhibited stable, surface-specific morpho-developmental adjustments. The core findings demonstrate that under water deficit, rice plants optimize their epidermal layer by modifying guard cell dimensions rather than shifting numerical frequency. Stomatal density remained numerically stable across moisture regimes, whereas cell dimensions displayed high plasticity. This plastic downsizing driven by sharp reductions in stomatal area and length was prominently expressed on the abaxial epidermis, which acts as the primary barrier against transpirational water loss.

Multi-environment analysis confirms that while grain yield is highly vulnerable to seasonal masking under stress, abaxial stomatal density and area maintain exceptionally robust genetic control across years. The persistent inverse trade-off between stomatal frequency and cell size remains stable across moisture treatments, confirming tight genetic regulation. Because stomatal features operate as upstream components of stress resilience rather than direct drivers of sink capacity, they display weak direct correlations with final grain yield under stress.

Operationally, breeders can focus exclusively on abaxial stomatal density under well-watered control conditions as a unified proxy. Selecting under control blocks successfully captures constitutive genetic variation while completely avoiding the severe leaf rolling and tissue deformations that distort slide impressions in drought plots. Integrating this phenotyping shortcut with deep-learning neural network architectures offers a high-throughput pathway to accelerate the development of climate-resilient rice cultivars.

## Data Availability

The raw data supporting the conclusions of this article will be made available by the authors, without undue reservation.
